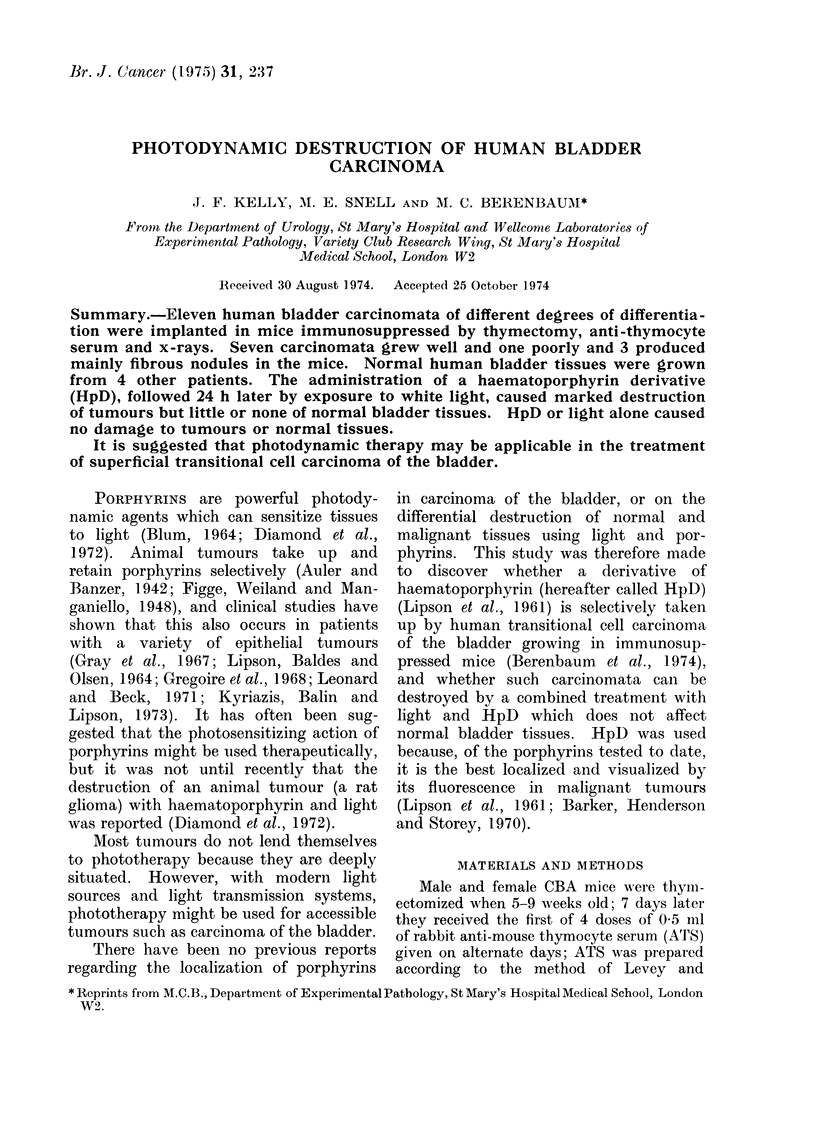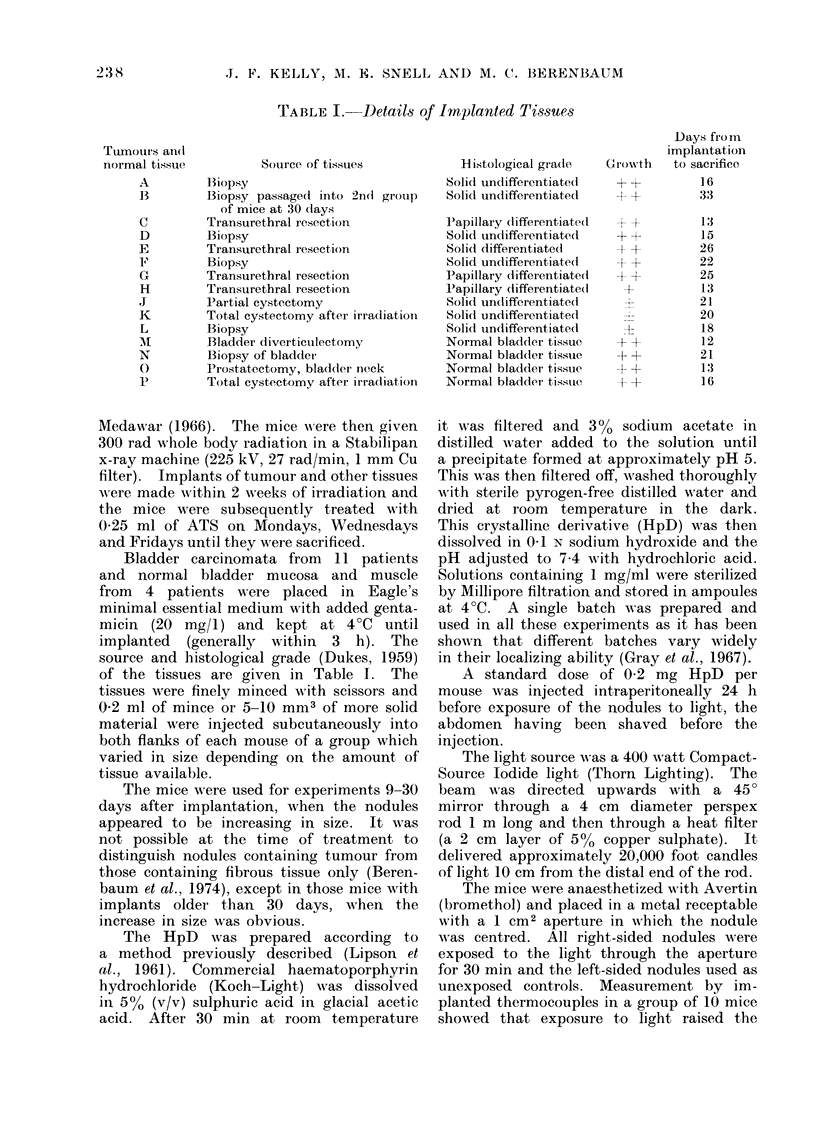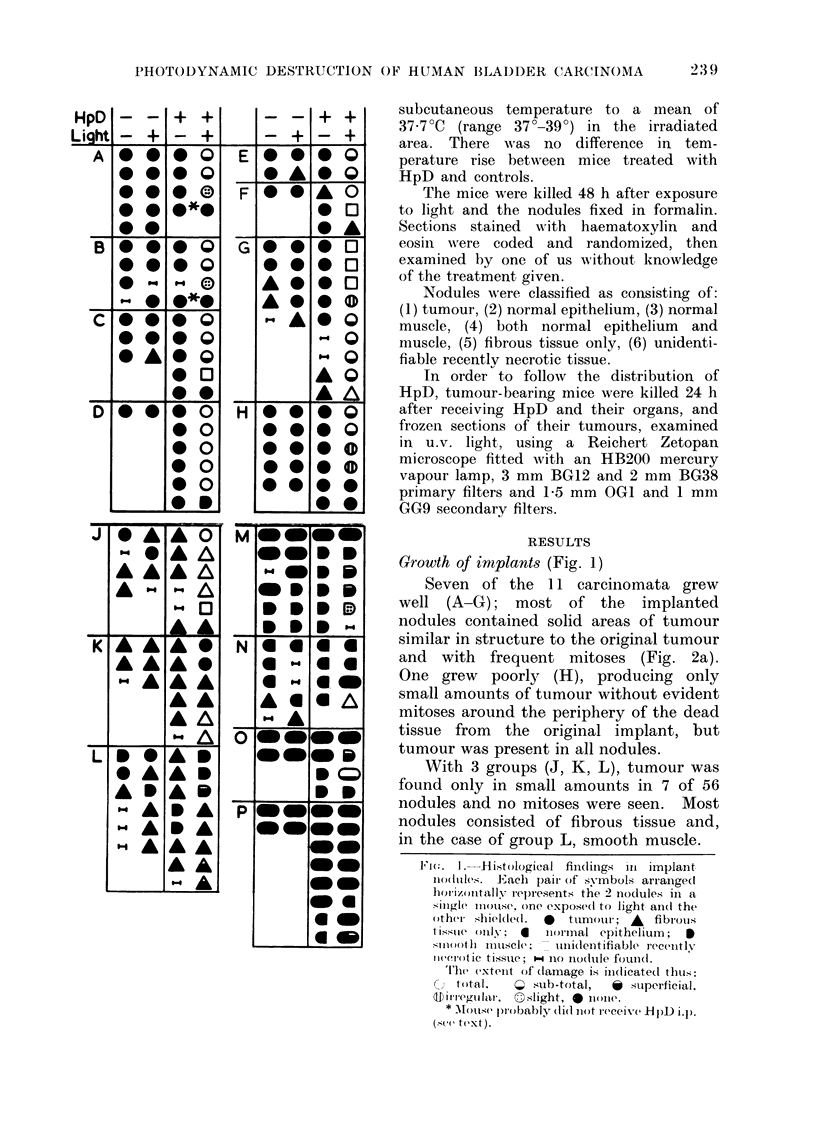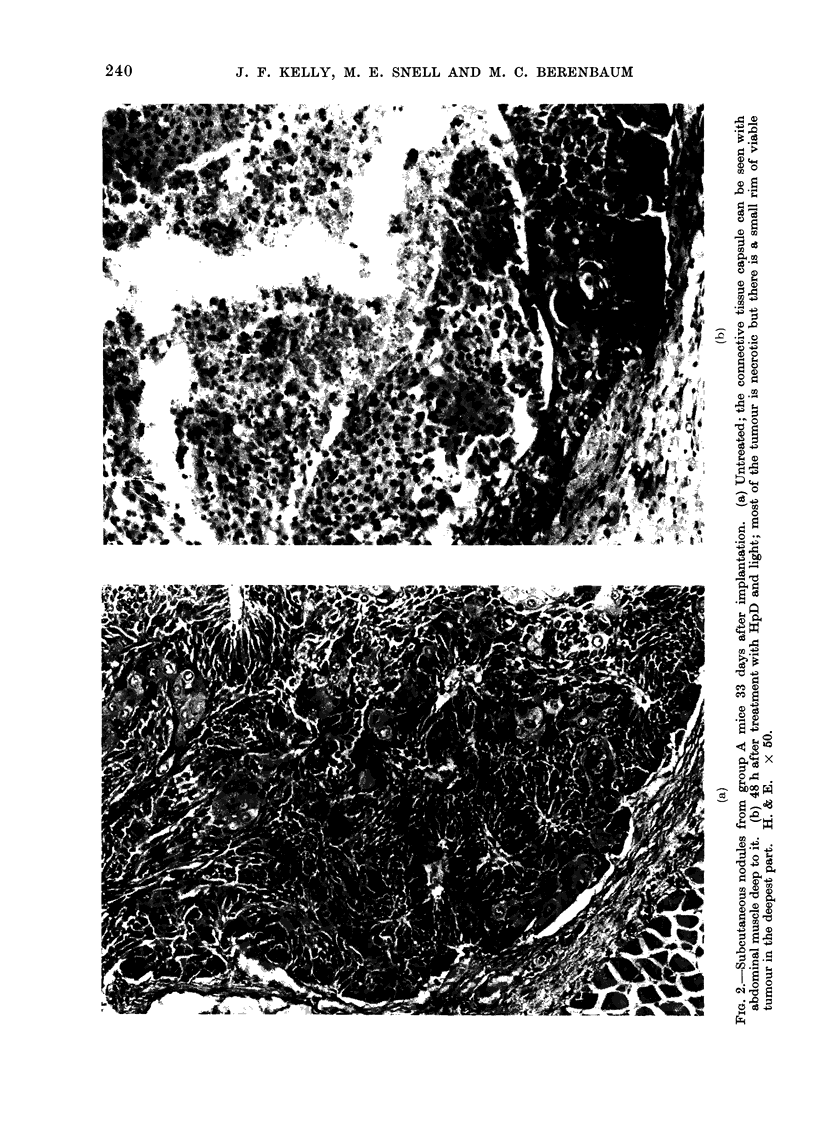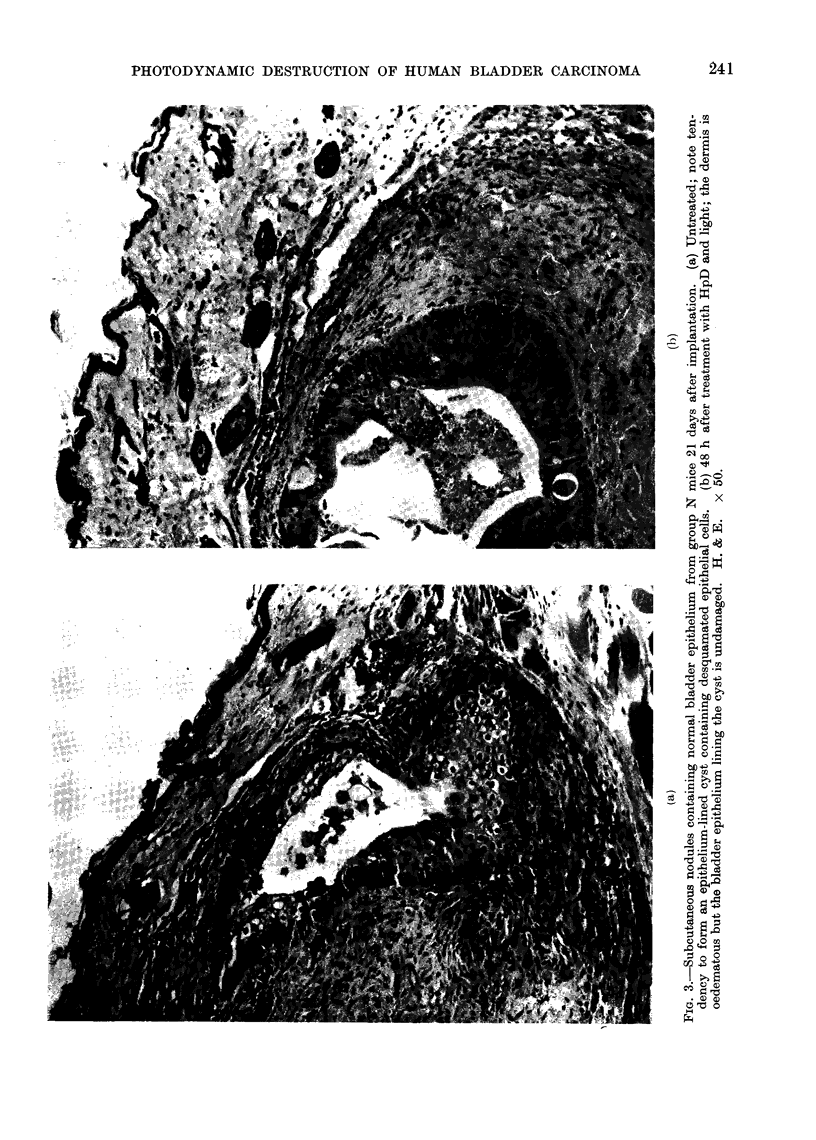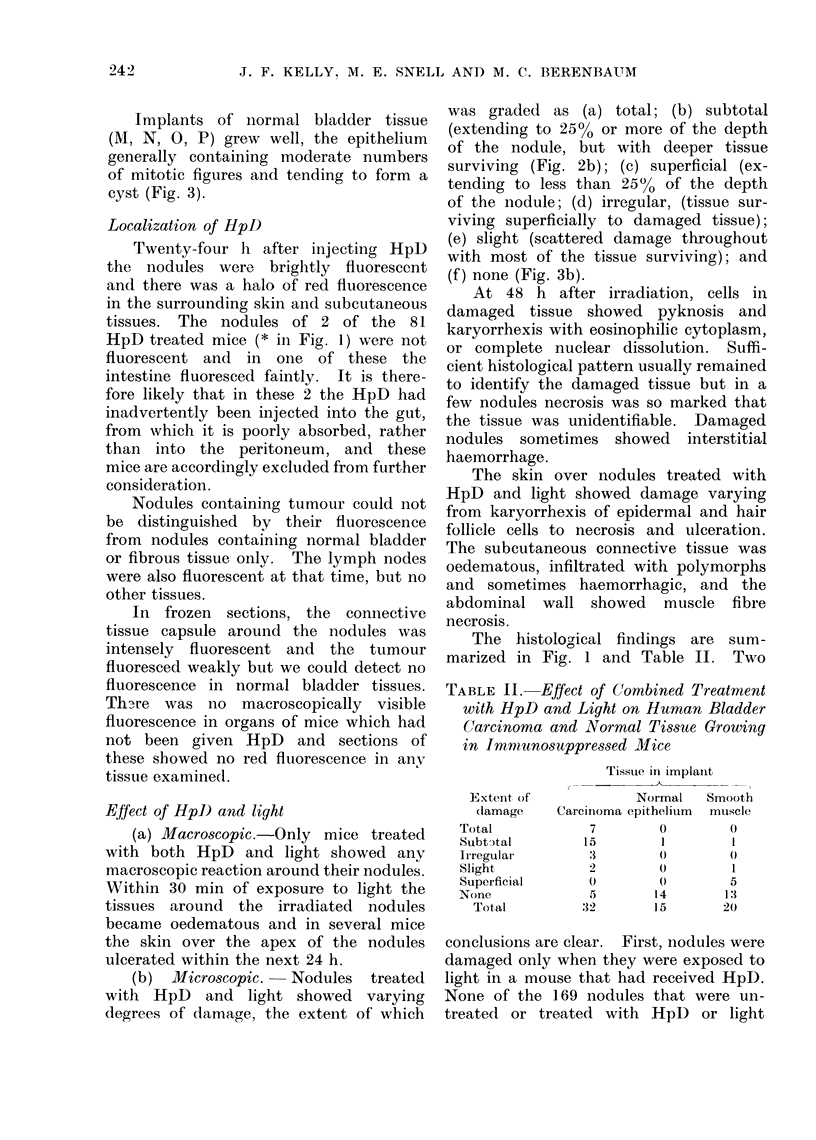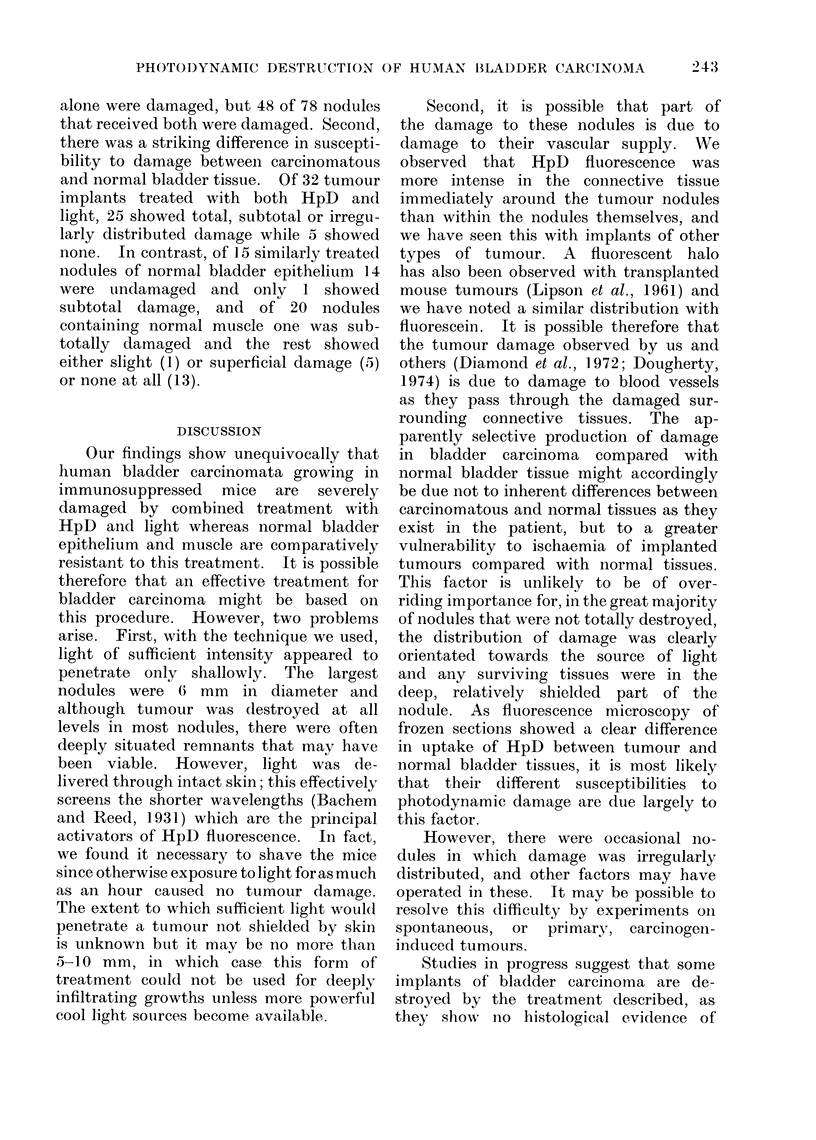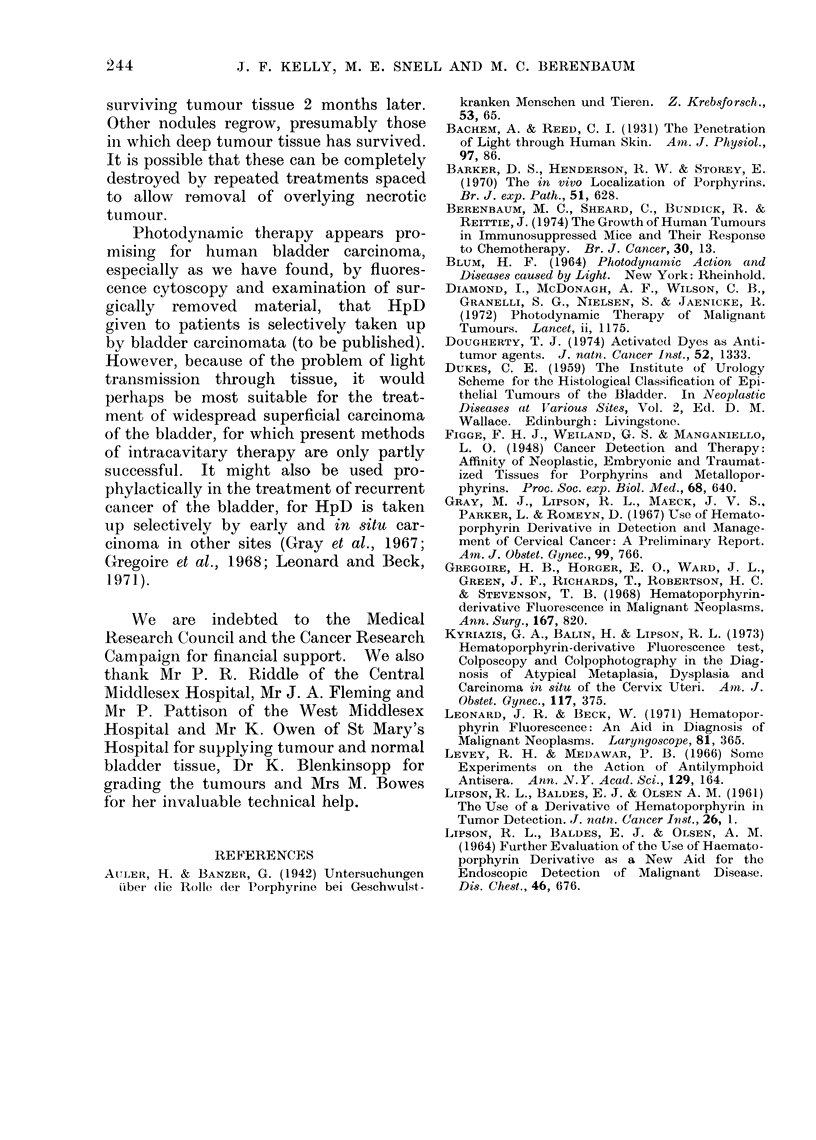# Photodynamic destruction of human bladder carcinoma.

**DOI:** 10.1038/bjc.1975.30

**Published:** 1975-02

**Authors:** J. F. Kelly, M. E. Snell, M. C. Berenbaum

## Abstract

**Images:**


*Br. J. Cancer* (1975) **31**, 237

### PHOTODYNAMIC DESTRUCTION OF HUMAN BLADDER CARCINOMA

J. F. KELLY, M. E. SNELL AND M. C. BERENBAUM*

*From the Department of Urology, St Mary's Hospital and Wellcome Laboratories of Experimental Pathology, Variety Club Research Wing, St Mary's Hospital Medical School, London W2*

Received 30 August 1974. Accepted 25 October 1974

**Summary.—Eleven human bladder carcinomata of different degrees of differentiation were implanted in mice immunosuppressed by thymectomy, anti-thymocyte serum and x-rays. Seven carcinomata grew well and one poorly and 3 produced mainly fibrous nodules in the mice. Normal human bladder tissues were grown from 4 other patients. The administration of a haematoporphyrin derivative (HpD), followed 24 h later by exposure to white light, caused marked destruction of tumours but little or none of normal bladder tissues. HpD or light alone caused no damage to tumours or normal tissues.**

**It is suggested that photodynamic therapy may be applicable in the treatment of superficial transitional cell carcinoma of the bladder.**

PORPHYRINS are powerful photodynamic agents which can sensitize tissues to light (Blum, 1964; Diamond *et al.*, 1972). Animal tumours take up and retain porphyrins selectively (Auler and Banzer, 1942; Figge, Weiland and Manganiello, 1948), and clinical studies have shown that this also occurs in patients with a variety of epithelial tumours (Gray *et al.*, 1967; Lipson, Baldes and Olsen, 1964; Gregoire *et al.*, 1968; Leonard and Beck, 1971; Kyriazis, Balin and Lipson, 1973). It has often been suggested that the photosensitizing action of porphyrins might be used therapeutically, but it was not until recently that the destruction of an animal tumour (a rat glioma) with haematoporphyrin and light was reported (Diamond *et al.*, 1972).

Most tumours do not lend themselves to phototherapy because they are deeply situated. However, with modern light sources and light transmission systems, phototherapy might be used for accessible tumours such as carcinoma of the bladder.

There have been no previous reports regarding the localization of porphyrins in carcinoma of the bladder, or on the differential destruction of normal and malignant tissues using light and porphyrins. This study was therefore made to discover whether a derivative of haematoporphyrin (hereafter called HpD) (Lipson *et al.*, 1961) is selectively taken up by human transitional cell carcinoma of the bladder growing in immunosuppressed mice (Berenbaum *et al.*, 1974), and whether such carcinomata can be destroyed by a combined treatment with light and HpD which does not affect normal bladder tissues. HpD was used because, of the porphyrins tested to date, it is the best localized and visualized by its fluorescence in malignant tumours (Lipson *et al.*, 1961; Barker, Henderson and Storey, 1970).

### MATERIALS AND METHODS

Male and female CBA mice were thymectomized when 5–9 weeks old; 7 days later they received the first of 4 doses of 0·5 ml of rabbit anti-mouse thymocyte serum (ATS) given on alternate days; ATS was prepared according to the method of Levey and

* Reprints from M.C.B., Department of Experimental Pathology, St Mary's Hospital Medical School, London W2.

TABLE I.—*Details of Implanted Tissues*

| Tumours and normal tissue | Source of tissues | Histological grade | Growth | Days from implantation to sacrifice |
|---|---|---|---|---|
| A | Biopsy | Solid undifferentiated | ++ | 16 |
| B | Biopsy passaged into 2nd group of mice at 30 days | Solid undifferentiated | ++ | 33 |
| C | Transurethral resection | Papillary differentiated | ++ | 13 |
| D | Biopsy | Solid undifferentiated | ++ | 15 |
| E | Transurethral resection | Solid differentiated | ++ | 26 |
| F | Biopsy | Solid undifferentiated | ++ | 22 |
| G | Transurethral resection | Papillary differentiated | ++ | 25 |
| H | Transurethral resection | Papillary differentiated | + | 13 |
| J | Partial cystectomy | Solid undifferentiated | ± | 21 |
| K | Total cystectomy after irradiation | Solid undifferentiated | ± | 20 |
| L | Biopsy | Solid undifferentiated | ± | 18 |
| M | Bladder diverticulectomy | Normal bladder tissue | ++ | 12 |
| N | Biopsy of bladder | Normal bladder tissue | ++ | 21 |
| O | Prostatectomy, bladder neck | Normal bladder tissue | ++ | 13 |
| P | Total cystectomy after irradiation | Normal bladder tissue | ++ | 16 |

Medawar (1966). The mice were then given 300 rad whole body radiation in a Stabilipan x-ray machine (225 kV, 27 rad/min, 1 mm Cu filter). Implants of tumour and other tissues were made within 2 weeks of irradiation and the mice were subsequently treated with 0·25 ml of ATS on Mondays, Wednesdays and Fridays until they were sacrificed.

Bladder carcinomata from 11 patients and normal bladder mucosa and muscle from 4 patients were placed in Eagle's minimal essential medium with added gentamicin (20 mg/l) and kept at 4°C until implanted (generally within 3 h). The source and histological grade (Dukes, 1959) of the tissues are given in Table I. The tissues were finely minced with scissors and 0·2 ml of mince or 5–10 $mm^3$ of more solid material were injected subcutaneously into both flanks of each mouse of a group which varied in size depending on the amount of tissue available.

The mice were used for experiments 9–30 days after implantation, when the nodules appeared to be increasing in size. It was not possible at the time of treatment to distinguish nodules containing tumour from those containing fibrous tissue only (Berenbaum *et al.*, 1974), except in those mice with implants older than 30 days, when the increase in size was obvious.

The HpD was prepared according to a method previously described (Lipson *et al.*, 1961). Commercial haematoporphyrin hydrochloride (Koch–Light) was dissolved in 5% (v/v) sulphuric acid in glacial acetic acid. After 30 min at room temperature it was filtered and 3% sodium acetate in distilled water added to the solution until a precipitate formed at approximately pH 5. This was then filtered off, washed thoroughly with sterile pyrogen-free distilled water and dried at room temperature in the dark. This crystalline derivative (HpD) was then dissolved in 0·1 N sodium hydroxide and the pH adjusted to 7·4 with hydrochloric acid. Solutions containing 1 mg/ml were sterilized by Millipore filtration and stored in ampoules at 4°C. A single batch was prepared and used in all these experiments as it has been shown that different batches vary widely in their localizing ability (Gray *et al.*, 1967).

A standard dose of 0·2 mg HpD per mouse was injected intraperitoneally 24 h before exposure of the nodules to light, the abdomen having been shaved before the injection.

The light source was a 400 watt Compact-Source Iodide light (Thorn Lighting). The beam was directed upwards with a 45° mirror through a 4 cm diameter perspex rod 1 m long and then through a heat filter (a 2 cm layer of 5% copper sulphate). It delivered approximately 20,000 foot candles of light 10 cm from the distal end of the rod.

The mice were anaesthetized with Avertin (bromethol) and placed in a metal receptable with a 1 $cm^2$ aperture in which the nodule was centred. All right-sided nodules were exposed to the light through the aperture for 30 min and the left-sided nodules used as unexposed controls. Measurement by implanted thermocouples in a group of 10 mice showed that exposure to light raised the subcutaneous temperature to a mean of 37·7 °C (range 37°–39°) in the irradiated area. There was no difference in temperature rise between mice treated with HpD and controls.

The mice were killed 48 h after exposure to light and the nodules fixed in formalin. Sections stained with haematoxylin and eosin were coded and randomized, then examined by one of us without knowledge of the treatment given.

Nodules were classified as consisting of: (1) tumour, (2) normal epithelium, (3) normal muscle, (4) both normal epithelium and muscle, (5) fibrous tissue only, (6) unidentifiable recently necrotic tissue.

In order to follow the distribution of HpD, tumour-bearing mice were killed 24 h after receiving HpD and their organs, and frozen sections of their tumours, examined in u.v. light, using a Reichert Zetopan microscope fitted with an HB200 mercury vapour lamp, 3 mm BG12 and 2 mm BG38 primary filters and 1·5 mm OG1 and 1 mm GG9 secondary filters.

### RESULTS

*Growth of implants* (Fig. 1)

Seven of the 11 carcinomata grew well (A–G); most of the implanted nodules contained solid areas of tumour similar in structure to the original tumour and with frequent mitoses (Fig. 2a). One grew poorly (H), producing only small amounts of tumour without evident mitoses around the periphery of the dead tissue from the original implant, but tumour was present in all nodules.

With 3 groups (J, K, L), tumour was found only in small amounts in 7 of 56 nodules and no mitoses were seen. Most nodules consisted of fibrous tissue and, in the case of group L, smooth muscle.

FIG. 1.—Histological findings in implant nodules. Each pair of symbols arranged horizontally represents the 2 nodules in a single mouse, one exposed to light and the other shielded. ● tumour; ▲ fibrous tissue only; ◖ normal epithelium; ◗ smooth muscle; ⬭ unidentifiable recently necrotic tissue; ⟷ no nodule found.

The extent of damage is indicated thus: ○ total, ◒ sub-total, ◓ superficial, ◍ irregular, ☺ slight, ● none.

* Mouse probably did not receive HpD i.p. (see text).

Implants of normal bladder tissue (M, N, O, P) grew well, the epithelium generally containing moderate numbers of mitotic figures and tending to form a cyst (Fig. 3).

*Localization of HpD*

Twenty-four h after injecting HpD the nodules were brightly fluorescent and there was a halo of red fluorescence in the surrounding skin and subcutaneous tissues. The nodules of 2 of the 81 HpD treated mice (* in Fig. 1) were not fluorescent and in one of these the intestine fluoresced faintly. It is therefore likely that in these 2 the HpD had inadvertently been injected into the gut, from which it is poorly absorbed, rather than into the peritoneum, and these mice are accordingly excluded from further consideration.

Nodules containing tumour could not be distinguished by their fluorescence from nodules containing normal bladder or fibrous tissue only. The lymph nodes were also fluorescent at that time, but no other tissues.

In frozen sections, the connective tissue capsule around the nodules was intensely fluorescent and the tumour fluoresced weakly but we could detect no fluorescence in normal bladder tissues. There was no macroscopically visible fluorescence in organs of mice which had not been given HpD and sections of these showed no red fluorescence in any tissue examined.

*Effect of HpD and light*

(a) *Macroscopic.*—Only mice treated with both HpD and light showed any macroscopic reaction around their nodules. Within 30 min of exposure to light the tissues around the irradiated nodules became oedematous and in several mice the skin over the apex of the nodules ulcerated within the next 24 h.

(b) *Microscopic.* — Nodules treated with HpD and light showed varying degrees of damage, the extent of which was graded as (a) total; (b) subtotal (extending to 25% or more of the depth of the nodule, but with deeper tissue surviving (Fig. 2b); (c) superficial (extending to less than 25% of the depth of the nodule; (d) irregular, (tissue surviving superficially to damaged tissue); (e) slight (scattered damage throughout with most of the tissue surviving); and (f) none (Fig. 3b).

At 48 h after irradiation, cells in damaged tissue showed pyknosis and karyorrhexis with eosinophilic cytoplasm, or complete nuclear dissolution. Sufficient histological pattern usually remained to identify the damaged tissue but in a few nodules necrosis was so marked that the tissue was unidentifiable. Damaged nodules sometimes showed interstitial haemorrhage.

The skin over nodules treated with HpD and light showed damage varying from karyorrhexis of epidermal and hair follicle cells to necrosis and ulceration. The subcutaneous connective tissue was oedematous, infiltrated with polymorphs and sometimes haemorrhagic, and the abdominal wall showed muscle fibre necrosis.

The histological findings are summarized in Fig. 1 and Table II. Two

TABLE II.—*Effect of Combined Treatment with HpD and Light on Human Bladder Carcinoma and Normal Tissue Growing in Immunosuppressed Mice*

| | Tissue in implant | | |
|---|---|---|---|
| Extent of damage | Carcinoma | Normal epithelium | Smooth muscle |
| Total | 7 | 0 | 0 |
| Subtotal | 15 | 1 | 1 |
| Irregular | 3 | 0 | 0 |
| Slight | 2 | 0 | 1 |
| Superficial | 0 | 0 | 5 |
| None | 5 | 14 | 13 |
| Total | 32 | 15 | 20 |

conclusions are clear. First, nodules were damaged only when they were exposed to light in a mouse that had received HpD. None of the 169 nodules that were untreated or treated with HpD or light alone were damaged, but 48 of 78 nodules that received both were damaged. Second, there was a striking difference in susceptibility to damage between carcinomatous and normal bladder tissue. Of 32 tumour implants treated with both HpD and light, 25 showed total, subtotal or irregularly distributed damage while 5 showed none. In contrast, of 15 similarly treated nodules of normal bladder epithelium 14 were undamaged and only 1 showed subtotal damage, and of 20 nodules containing normal muscle one was subtotally damaged and the rest showed either slight (1) or superficial damage (5) or none at all (13).

### DISCUSSION

Our findings show unequivocally that human bladder carcinomata growing in immunosuppressed mice are severely damaged by combined treatment with HpD and light whereas normal bladder epithelium and muscle are comparatively resistant to this treatment. It is possible therefore that an effective treatment for bladder carcinoma might be based on this procedure. However, two problems arise. First, with the technique we used, light of sufficient intensity appeared to penetrate only shallowly. The largest nodules were 6 mm in diameter and although tumour was destroyed at all levels in most nodules, there were often deeply situated remnants that may have been viable. However, light was delivered through intact skin; this effectively screens the shorter wavelengths (Bachem and Reed, 1931) which are the principal activators of HpD fluorescence. In fact, we found it necessary to shave the mice since otherwise exposure to light for as much as an hour caused no tumour damage. The extent to which sufficient light would penetrate a tumour not shielded by skin is unknown but it may be no more than 5–10 mm, in which case this form of treatment could not be used for deeply infiltrating growths unless more powerful cool light sources become available.

Second, it is possible that part of the damage to these nodules is due to damage to their vascular supply. We observed that HpD fluorescence was more intense in the connective tissue immediately around the tumour nodules than within the nodules themselves, and we have seen this with implants of other types of tumour. A fluorescent halo has also been observed with transplanted mouse tumours (Lipson *et al.*, 1961) and we have noted a similar distribution with fluorescein. It is possible therefore that the tumour damage observed by us and others (Diamond *et al.*, 1972; Dougherty, 1974) is due to damage to blood vessels as they pass through the damaged surrounding connective tissues. The apparently selective production of damage in bladder carcinoma compared with normal bladder tissue might accordingly be due not to inherent differences between carcinomatous and normal tissues as they exist in the patient, but to a greater vulnerability to ischaemia of implanted tumours compared with normal tissues. This factor is unlikely to be of overriding importance for, in the great majority of nodules that were not totally destroyed, the distribution of damage was clearly orientated towards the source of light and any surviving tissues were in the deep, relatively shielded part of the nodule. As fluorescence microscopy of frozen sections showed a clear difference in uptake of HpD between tumour and normal bladder tissues, it is most likely that their different susceptibilities to photodynamic damage are due largely to this factor.

However, there were occasional nodules in which damage was irregularly distributed, and other factors may have operated in these. It may be possible to resolve this difficulty by experiments on spontaneous, or primary, carcinogen-induced tumours.

Studies in progress suggest that some implants of bladder carcinoma are destroyed by the treatment described, as they show no histological evidence of surviving tumour tissue 2 months later. Other nodules regrow, presumably those in which deep tumour tissue has survived. It is possible that these can be completely destroyed by repeated treatments spaced to allow removal of overlying necrotic tumour.

Photodynamic therapy appears promising for human bladder carcinoma, especially as we have found, by fluorescence cytoscopy and examination of surgically removed material, that HpD given to patients is selectively taken up by bladder carcinomata (to be published). However, because of the problem of light transmission through tissue, it would perhaps be most suitable for the treatment of widespread superficial carcinoma of the bladder, for which present methods of intracavitary therapy are only partly successful. It might also be used prophylactically in the treatment of recurrent cancer of the bladder, for HpD is taken up selectively by early and *in situ* carcinoma in other sites (Gray *et al.*, 1967; Gregoire *et al.*, 1968; Leonard and Beck, 1971).

We are indebted to the Medical Research Council and the Cancer Research Campaign for financial support. We also thank Mr P. R. Riddle of the Central Middlesex Hospital, Mr J. A. Fleming and Mr P. Pattison of the West Middlesex Hospital and Mr K. Owen of St Mary's Hospital for supplying tumour and normal bladder tissue, Dr K. Blenkinsopp for grading the tumours and Mrs M. Bowes for her invaluable technical help.

### REFERENCES

AULER, H. & BANZER, G. (1942) Untersuchungen über die Rolle der Porphyrine bei Geschwulstkranken Menschen und Tieren. *Z. Krebsforsch.*, **53**, 65.

BACHEM, A. & REED, C. I. (1931) The Penetration of Light through Human Skin. *Am. J. Physiol.*, **97**, 86.

BARKER, D. S., HENDERSON, R. W. & STOREY, E. (1970) The *in vivo* Localization of Porphyrins. *Br. J. exp. Path.*, **51**, 628.

BERENBAUM, M. C., SHEARD, C., BUNDICK, R. & REITTIE, J. (1974) The Growth of Human Tumours in Immunosuppressed Mice and Their Response to Chemotherapy. *Br. J. Cancer*, **30**, 13.

BLUM, H. F. (1964) *Photodynamic Action and Diseases caused by Light.* New York: Rheinhold.

DIAMOND, I., McDONAGH, A. F., WILSON, C. B., GRANELLI, S. G., NIELSEN, S. & JAENICKE, R. (1972) Photodynamic Therapy of Malignant Tumours. *Lancet*, ii, 1175.

DOUGHERTY, T. J. (1974) Activated Dyes as Antitumor agents. *J. natn. Cancer Inst.*, **52**, 1333.

DUKES, C. E. (1959) The Institute of Urology Scheme for the Histological Classification of Epithelial Tumours of the Bladder. In *Neoplastic Diseases at Various Sites*, Vol. 2, Ed. D. M. Wallace. Edinburgh: Livingstone.

FIGGE, F. H. J., WEILAND, G. S. & MANGANIELLO, L. O. (1948) Cancer Detection and Therapy: Affinity of Neoplastic, Embryonic and Traumatized Tissues for Porphyrins and Metalloporphyrins. *Proc. Soc. exp. Biol. Med.*, **68**, 640.

GRAY, M. J., LIPSON, R. L., MAECK, J. V. S., PARKER, L. & ROMEYN, D. (1967) Use of Hematoporphyrin Derivative in Detection and Management of Cervical Cancer: A Preliminary Report. *Am. J. Obstet. Gynec.*, **99**, 766.

GREGOIRE, H. B., HORGER, E. O., WARD, J. L., GREEN, J. F., RICHARDS, T., ROBERTSON, H. C. & STEVENSON, T. B. (1968) Hematoporphyrin-derivative Fluorescence in Malignant Neoplasms. *Ann. Surg.*, **167**, 820.

KYRIAZIS, G. A., BALIN, H. & LIPSON, R. L. (1973) Hematoporphyrin-derivative Fluorescence test, Colposcopy and Colpophotography in the Diagnosis of Atypical Metaplasia, Dysplasia and Carcinoma *in situ* of the Cervix Uteri. *Am. J. Obstet. Gynec.*, **117**, 375.

LEONARD, J. R. & BECK, W. (1971) Hematoporphyrin Fluorescence: An Aid in Diagnosis of Malignant Neoplasms. *Laryngoscope*, **81**, 365.

LEVEY, R. H. & MEDAWAR, P. B. (1966) Some Experiments on the Action of Antilymphoid Antisera. *Ann. N.Y. Acad. Sci.*, **129**, 164.

LIPSON, R. L., BALDES, E. J. & OLSEN A. M. (1961) The Use of a Derivative of Hematoporphyrin in Tumor Detection. *J. natn. Cancer Inst.*, **26**, 1.

LIPSON, R. L., BALDES, E. J. & OLSEN, A. M. (1964) Further Evaluation of the Use of Haematoporphyrin Derivative as a New Aid for the Endoscopic Detection of Malignant Disease. *Dis. Chest.*, **46**, 676.